# *LINC01116* affects patient survival differently and is dissimilarly expressed in ER+ and ER− breast cancer samples

**DOI:** 10.1002/cnr2.1848

**Published:** 2023-06-15

**Authors:** Mohammadjavad Karimi Taheri, Sogol Ghanbari, Akram Gholipour, Taraneh Givi, Majid Sadeghizadeh

**Affiliations:** ^1^ Molecular Genetics Department Biological Sciences Faculty, Tarbiat Modares University Tehran Iran; ^2^ Cardiogenetic Research Center, Rajaie Cardiovascular Medical and Research Center Iran University of Medical Sciences Tehran Iran

**Keywords:** breast cancer, estrogen receptor, *LINC01116*, lncRNA, TGF‐β

## Abstract

**Background:**

Breast cancer is the most commonly detected cancer and one of the leading causes of cancer mortality. Emerging evidence supports that aberrant expression of lncRNAs is correlated with tumor progression and various aspects of tumor development.

**Aim:**

This study aimed to evaluate the expression pattern of *LINC01116* in breast cancer tissues and investigate the impact of *LINC01116* on patients' survival.

**Methods and Results:**

Microarray and qRT‐PCR data analysis were performed, and the KM‐plotter database was used in this study. In addition, the gain of function approach was performed to examine the effect of *LINC01116* on breast cancer cells in‐vitro. The results exhibited that *LINC01116* is meaningfully upregulated in the ER+ tumor specimens compared to the ER– ones. Also, relative to normal tissues, the expression of *LINC01116* in ER+ and ER– tumor tissues significantly increased and decreased, respectively. ROC curve analysis revealed the power of *LINC01116* in distinguishing ER+ from ER– samples. Additionally, the Kaplan‐Meier survival analysis showed that the *LINC01116* expression positively correlates with survival probability in all as well as ER+ patients. However, this correlation was negative in ER– patients. Furthermore, our results showed that the overexpression of *LINC01116* induces TGF‐β signaling in ER– cells (MDA‐MB‐231), and microarray data analysis revealed that *LINC01116* is significantly upregulated in 17β‐Estradiol treated MCF7 cells.

**Conclusion:**

In conclusion, our results suggest that *LINC01116* can be a potential biomarker in distinguishing ER+ and ER– tissues and has different effects on patients' survival based on ER status by affecting TGF‐β and ER signaling.

## 1 INTRODUCTION

Breast cancer, with a global prevalence of approximately 2.2 million cases in 2020, is the most common cancer worldwide and the leading cause of death among women, with about 685 000 cases, which accounted for about 15.5% of all cancers in 2020.^1^, ^2^ Early detection of breast cancer is a critical factor in preventing its development and metastasis, as well as reducing the mortality rate associated with the disease.^3^, ^4^ Hence, identifying potential molecular targets, regulatory elements, and diagnostic and prognostic biomarkers, such as various non‐coding RNAs (ncRNA), could be helpful in cancer prevention and therapy.^5^, ^6^

Non‐coding RNAs have emerged as a crucial player in cancer progression and inhibition of tumorigenesis. Recent studies have shown that ncRNAs, such as microRNAs, long non‐coding RNAs (lncRNAs), and circular RNAs, can regulate gene expression at different levels of the central dogma of molecular biology, affecting various cellular processes and cancer development, such as tumor growth, angiogenesis, cell cycle, drug resistance, and epithelial to mesenchymal transition (EMT).^7^, ^8^ Moreover, ncRNAs have gained significant attention as potential diagnostic biomarkers for cancer due to their tissue‐specific expression patterns and stability in body fluids. Identifying specific ncRNA signatures associated with different types of cancer could lead to the development of non‐invasive diagnostic tools for early detection and monitoring of disease progression. The study of ncRNA has opened up new avenues for understanding the molecular mechanisms underlying cancer pathogenesis and has provided promising targets for therapeutic intervention.^9^, ^10^

Breast cancer, as a heterogeneous malignancy, should not be considered a single disease and can be classified into different major subtypes based on Estrogen receptor (ER) status: Estrogen‐receptor‐positive (ER+) and Estrogen‐receptor‐negative (ER−), which ER+ accounts for about 70% of breast cancer.^11^ Detection of ER+ or ER− in breast cancer can allow for timely treatment and management of the disease before it progresses to an advanced stage. In addition, knowing the subtype of breast cancer can help healthcare providers tailor treatment plans to each patient's needs.^12^

Although it is well‐reported that ER signaling involves many mitogenic roles, such as cell growth, proliferation, and anti‐apoptotic effects,^13^, ^14^ ER signaling maintains the epithelial phenotype and opposes EMT.^14^, ^15^ In contrast, transforming growth factor‐β (TGF‐β) oppose these roles; it can induce EMT and reveals an antiproliferative effect in breast cancer cells. Interestingly, there is a close crosstalk between ER and TGF‐β, and they can suppress each other's signaling. TGF‐β signaling correlates with breast tumors and poor prognosis. In addition, TGF‐induced migration and invasion of breast cancer cells are reduced by ER signaling activation.^16^ It should be emphasized that several studies have reported that lncRNAs are involved in TGF‐β signaling in various cancers.^17^

*LINC01116* (long intergenic non‐protein coding RNA 1116), also known as *TALNEC2* as well termed ENSG00000163364 [Ensembl], is a 1058 bp lncRNA located in the 2q31.1 genomic region.^18^ *LINC01116* functions in proliferation, apoptosis, and cell cycle, as an oncogene have been investigated in various cancers, such as glioma,^19^, ^20^ lung adenocarcinoma,^21^, ^22^ prostate cancer,^23^ and breast cancer.^24^ The expression of *LINC01116* is significantly upregulated in all these cancers.^19^, ^20^, ^21^, ^22^, ^23^, ^24^ *LINC01116* has also been shown to exacerbate hypoxia or ischemic injuries in myocardial,^25^ cerebral ischemia,^26^ and osteonecrosis.^27^ It has been reported that *LINC01116* expression was significantly upregulated under hypoxia, causing apoptosis and decreasing cell viability, invasion, and migration in a cardiomyocyte's cell line (H9c2).^25^ It has been reported that the downregulation of *LINC01116* suppresses the AKT signaling pathway in lung adenocarcinoma.^21^ On the other hand, It has been proved that the overexpression of *LINC01116* caused inhibition of the PI3K/AKT/mTOR signaling pathway and decreased cell viability.^27^ Also, Cao Y et al. have demonstrated that *LINC01116* negatively regulates miR‐650 leading to an upregulation of APAF1 in Neuroblastoma cells and inducing cell apoptosis.^26^ Altogether, these findings suggest that the *LINC01116* plays a context‐dependent role.

In the present study using microarray analysis, we first identified the most significant differentially expressed lncRNAs in ER+ samples compared to ER− ones and found the *LINC01116* as the most significant upregulated lncRNA in ER+ samples for the first time. The receiver operating characteristic (ROC) curve analysis of the microarray and quantitative real‐time polymerase chain reaction (qRT‐PCR) data suggests that *LINC01116* can be a potential biomarker to distinguish ER+ from ER− breast cancer subtypes. The obtained results from this study suggest that the *LINC01116* expression differentially affects the survival rate in breast cancer patients based on ER status by affecting the ER and TGF‐β signaling pathways.

## 2 METHODS

### 2.1 Microarray data analysis

To investigate which lncRNA in ER+ breast samples is significantly upregulated compared to ER− ones and to identify the effect of ER signaling on the *LINC01116* expression, we used the microarray data analysis. A group of three available datasets, including microarray data from breast cancer samples, was used. Data sets were downloaded from gene expression omnibus (GEO)^28^, ^29^ with the identifiers GSE45827^30^ to examine the expression of *LINC01116* in breast cancer subtypes (luminal A = 29, luminal B = 30, triple‐negative breast cancer (TNBC) = 41, human epidermal growth factor receptor 2 (HER2) = 30, Normal = 11); GSE46924,^31^ in order to investigate the impact of 17β‐Estradiol treatment on *LINC01116* expression; and GSE26459^32^ to indicate the *LINC01116* expression in Tamoxifen resistance cells. These datasets were analyzed using the R language with the help of limma, GEOquery, and genefilter packages.^33^, ^34^, ^35^, ^36^ Quantile normalization and log2 transformation were used to modify the count data. Also, the false discovery rate (FDR) method was applied to calculate the adjusted *p*‐value (Adj. *p*‐value). The final graphs were produced by the pheatmap package^37^ and GraphPad Prism software version 8.0.1 (GraphPad Software Inc., USA).

### 2.2 Sample collection and RNA extraction

To identify the *LINC01116* expression pattern in breast cancer with qRT‐PCR, Primary breast tumor tissues were collected from patients at Rasoul‐Akram and Khatam‐al‐Anbia Hospitals in Tehran, Iran. The patients in this study had not yet received previous treatments, such as chemotherapy or radiotherapy. The use of pathological tissues was authorized by the Ferdowsi University of Mashhad Ethics Commission (IR.UM.REC.1399.104). The study was conducted in accordance with the guidelines outlined in the 1964 Helsinki Declaration. Signed written informed consent was provided by the patients after being informed about the purpose of the study. Forty pairs of breast tumors and their margin tissues were gathered from October 2018 to June 2019. The samples were analyzed by a pathologist and categorized based on the standard histopathological parameters. The samples were instantly snap‐frozen in liquid nitrogen and kept at −80°C until the RNA isolation process. The total RNA was extracted from all tissues using RiboEx Total RNA (GeneAll Biotechnology, South Korea (Cat No. 301‐001)). The quality of extracted RNAs was analyzed by The NanoDrop® ND‐1000 Spectrophotometer (Thermo Fisher Scientific., USA) and gel electrophoresis. The clinic pathological characteristics of patients are summarized in Table 1. In addition, details of sample information are presented in the supplementary material Table S1.

**TABLE 1:** Clinicopathologic features of breast cancer specimens.

Age (mean ± SD)	49.85 ± 10.3	TNM classification	Cases (n)
Tumor Subtype	**Cases (n)**	T = 1	14
Luminal A	26	T = 2	17
Luminal B	3	T = 3	3
Her2 Overexpressed	3	Undefined T	6
TNBC	6	N = 0	20
Undefined Subtype	2	N = 1	8
Grade	**Cases (n)**	N = 2	5
I	4	N = 3	1
II	21	Undefined N	6
III	10	M = 0	40
Undefined Grade	5		
Stage	**Cases (n)**		
I	9		
IIA	13		
IIB	7		
IIIA	4		
IIIC	1		
Undefined Stage	6		

Abbreviations: M, distant metastases (according to the TNM classification system); N, Lymph node status; T, tumor size; TNBC, Triple Negative Breast Cancer.

### 2.3 Cell culture

The breast cancer cell line, MDA‐MB‐231 was purchased from the National Cell Bank of Iran (Pasteur Institute, Iran (Cat No. C578)) and was cultured in dulbecco's modified eagle medium (DMEM) (Gibco, USA (Cat No. 11965084)) containing 10% fetal bovine serum (FBS) (Gibco, USA (Cat No. 16000044)) and 1% penicillin/streptomycin (10 000 units/mL of penicillin, 10 000 μg/mL of streptomycin; Gibco, USA (Cat No. 15140122)) at a 37°C incubator filling with 5% CO2.

### 2.4 Plasmid construction and cell transfection

To identify the effect of *LINC01116* overexpression on TGF‐β signaling in ER− breast cancer cells (MDA‐MB‐231), *LINC01116* was amplified with PCR and inserted into the pCDH‐CMV‐MCS‐EF1‐copGFP‐T2A‐Puro expression vector (System Biosciences, USA(Cat No. CD511B‐1)) at sites EcoRI and NotI restriction enzymes (New England Biolabs, USA (Cat No. R0101S (EcoRI) and R0189S (NotI))). The primers used for cloning are shown as *LINC01116* forward primer: 5’‐AAACCGGAATTCAGGAAATGACCCGAACTGC‐3′ and reverse primer: 5’‐ATAAGAATGCGGCCGCCATTCACGTATTCTTCCAGTGTCTT‐3′. MDA‐MB‐231 cells were seeded, at least 24 h before transfection, into a 24‐wells plate. When the cells density was about 70%, they were transfected with Mock (PCDH‐Mock) and *LINC01116* (PCDH‐LINC01116) vectors according to Lipofectamine™ 2000 (Invitrogen, USA (Cat No. 11668030)) manufacturer's instruction. Total RNA was extracted from the transfected cells after 48 h of incubation of the cells for qRT‐PCR.

### 2.5 Quantitative real‐time polymerase chain reaction

The quantitative real‐time polymerase chain reaction (qRT‐PCR) was performed to evaluate *LINC01116* expression in breast cancer samples. For this purpose, 2 μg of each extracted RNA sample was used for cDNA synthesis using the cDNA Synthesis Kit (Thermo Fisher Scientific., USA (Cat No. K1622)). For detecting the gene expression, qRT‐PCR was performed using a BIOFACT™ High ROX SYBR Green (BioFACT, South Korea (Cat No. DQ368‐40h)) in the StepOne Real‐Time PCR (Applied Biosystems, USA). Thermal cycling settings included a 5‐minute incubation at 95°C; followed by 40 cycles of 95°C for 30 s, 60°C for 30 s, and 72°C for 30 s; followed by a 5‐minute extension period at 72°C. Then the target genes were normalized to the housekeeping gene *β2m* as an internal control, and the fold changes of expression level were calculated using the relative quantification (2^−ΔΔCt^) method. The primers used in this study are listed in the supplementary material Table S2.

### 2.6 Survival analysis through Kaplan–Meier plot

We used the KM plotter, a web‐based tool (https://kmplot.com), to analyze the effect of gene expression on survival patients based on clinicopathological data, such as different hormone receptors, subtypes, and tumor grades.^38^ Sources for the database are GEO, the European genome‐phenome archive (EGA), and the cancer genome atlas program (TCGA), to mention but a handful. KM plotter explores the correlation between gene‐specific expression levels and relapse‐free survival (RFS) in breast cancer patients by applying the RNA‐seq and microarray data separately. However, we used both of them in this research.

### 2.7 KEGG pathway analysis of differentially correlated genes

The powerful, comprehensive, online enrichment tool (Enrichr^39^, ^40^, ^41^) was utilized to use the Kyoto encyclopedia of genes and genomes (KEGG) pathway analysis^42^ for the differentially correlated genes with *LINC01116 (Pearson correlation coefficient method*).

### 2.8 Statistical analysis

The results are presented as means ± standard deviation (SD). The student's *t*‐test was applied for gene expression evaluation in qRT‐PCR, using GraphPad Prism software version 8.0.1 (GraphPad Software Inc., USA). The ROC curve analysis was performed using the GraphPad Prism software version 8.0.1 (confidence interval: 95%, method: Wilson). In qRT‐PCR data analysis, *p*‐value <.05, and for microarray data analysis and KEGG pathway results, Adj. *p*‐value <.05 was considered statistically significant.

## 3 RESULTS

### 3.1 *LINC01116* is highly expressed in ER+ samples

**FIGURE 1:**
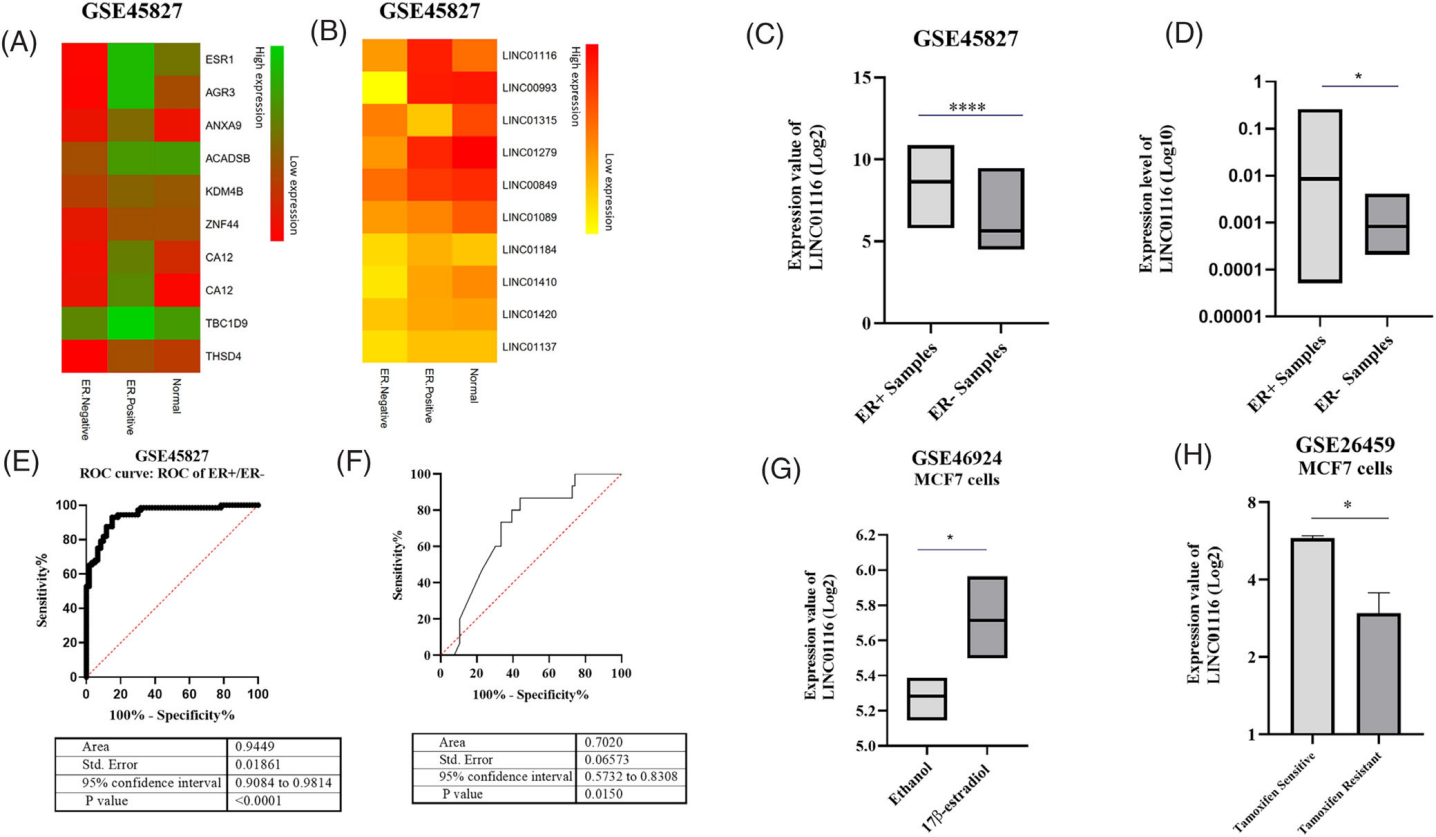
*LINC01116* is differentially expressed in ER+ and ER− breast samples. None‐clustered‐row heatmap of genes (A) and lncRNAs (B) differentially expressed in ER+ and ER− samples (59 ER+ and 71 ER− samples). GSE45827 microarray data analysis of *LINC01116* expression level in ER+ and ER− samples, box plots show the mean of overall RNA expression in ER+ and ER− samples. ****Adj. *p*‐value <.0001 (C). Quantitative RT‐PCR analysis of *LINC01116* expression level in ER+ and ER− tissues (29 ER+ and 11 ER− tissues), box plots show the mean of overall RNA expression in ER+ and ER− tissues.* *p*‐value <.05 (D). ROC curve shows the power of *LINC01116* in distinguishing ER+ from ER− in breast cancer samples (E and F). GSE46924 dataset analysis of *LINC01116* expression level in 17β‐Estradiol treated MCF7 cells; box plots show the mean of overall RNA expression in 17β‐Estradiol‐treated and Ethanol‐treated MCF7 cells. *Adj. *p*‐value <.05 (G). GSE26459 dataset analysis of *LINC01116* expression level in Tamoxifen resistance and sensitive MCF7 cells; box plots show the mean of overall RNA expression in Tamoxifen resistance and sensitive MCF7 cells. *Adj. *p*‐value <.05 (H).

Firstly, differentially expressed genes in ER+ and ER− samples were investigated by analyzing data from the GSE45827 dataset (Figure 1A). Then we applied a filter to recognize the top differentially expressed lncRNAs (Figure 1B), and *LINC01116* was the most significant lncRNA with a high expression level in the ER+ samples compared to the ER− ones (Log fold change (FC) ≈ 2.7) (Table 2 and Figure 1C). Furthermore, qRT‐PCR was performed on tumor and margin tumor breast tissues (40 pairs), and results showed a 10.3‐fold increase in *LINC01116* expression in ER+ compared with ER− tissues (Figure 1D). Additionally, to examine the power of *LINC01116* in distinguishing ER+ from ER− in breast cancer samples, the ROC curve was used to evaluate the sensitivity and specificity of *LINC01116*. The area under the curve of *LINC01116* was calculated at about 0.94 (*p*‐value <.0001) for GSE45827 samples (Figure 1E) and 0.7 (*p*‐value = .015) for breast cancer tissues (Figure 1F), which is deemed as a potential biomarker in ER+ breast cancer. In addition, using the GSE46924 microarray dataset, the impact of 17β‐Estradiol treatment on *LINC01116* expression was evaluated to ascertain the effect of Estrogen signaling on its expression. The result exhibited a significant increase in the expression of *LINC01116* in MCF7 cells treated with 17β‐Estradiol compared to the control (Ethanol) (Figure 1G). More inquiringly, the GSE26459 dataset was applied to investigate the effect of ligand‐independent Estrogen signaling (like Tamoxifen resistance cells)^43^ on the expression of *LINC01116*. Surprisingly, data showed the expression of *LINC01116* significantly downregulated in MCF7 Tamoxifen‐resistant cells compared to Tamoxifen‐sensitive cells (Figure 1H).

**TABLE 2:** Differentially expressed lncRNAs in ER+ and ER− breast samples.

Gene symbol	LogFC	adj.P.Val
***LINC01116***	**2.723110854**	**1.68E‐24**
*LINC00993*	5.277313825	4.84E‐16
*LINC01315*	−1.60600291	4.26E‐12
*LINC01279*	2.508342284	7.46E‐10
*LINC00849*	1.190609945	2.00E‐09
*LINC01089*	0.493671069	3.09E‐09
*LINC01184*	0.88423902	3.28E‐08
*LINC01410*	1.492082193	5.44E‐08
*LINC01420*	0.690373017	6.07E‐08
*LINC01137*	0.65533733	1.13E‐07

### 3.2 The *LINC01116* expression pattern in breast cancer tissues

**FIGURE 2:**
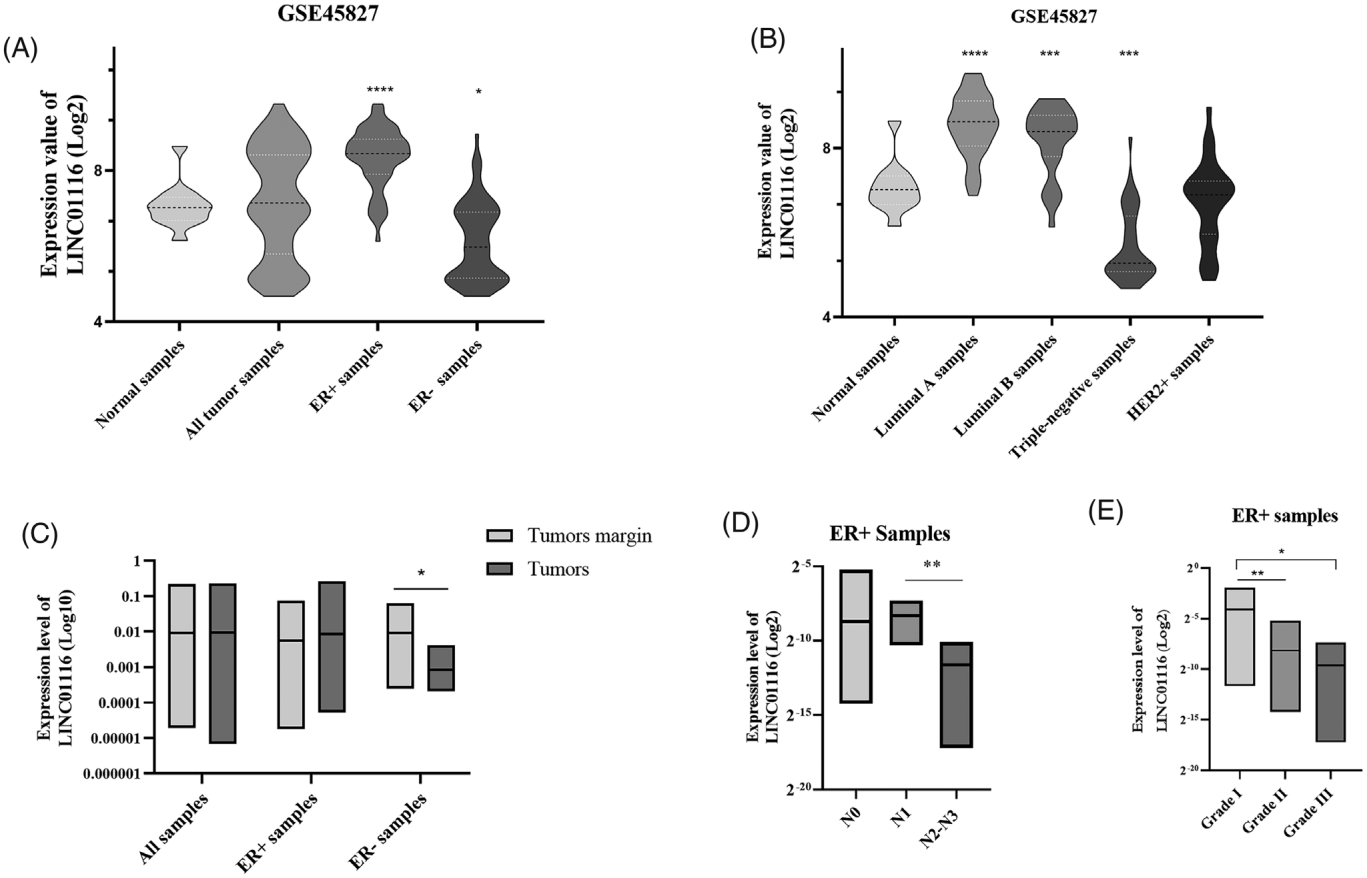
The alteration of the *LINC01116* expression level in breast cancer tissues. Violin plots of GSE45827 microarray data analysis show the mean of overall RNA expression in tumor, ER+, and ER− relative to normal samples. ****Adj. *p*‐value <.0001, *Adj. *p*‐value <.05 (A). Violin plots show the mean of overall RNA expression in Luminal A, Luminal B, TNBC, and HER2+ compared to normal samples based on GSE45827 microarray data analysis. ****Adj. *p*‐value <.0001, ***Adj. *p*‐value <.001 (B). Quantitative RT‐PCR analysis of *LINC01116* level in 40 paired (29 pairs of ER+ and 11 pairs ER−) breast cancer and tumor margin tissues; box plots represent the mean of overall RNA expression, * *p*‐value<.05 (C). Quantitative RT‐PCR analysis of *LINC01116* level in ER+ tissues; box plots show the mean of overall RNA expression in lymph nodes N0 (*n* = 15), N1 (*n* = 6), and N2 and N3 (*n* = 4), **p‐value<.01(D) and in grade I (*n* = 4), grade II (*n* = 18), and grade III (*n* = 5), *p‐value<.05, **p‐value<.01 (E).

We analyzed the expression of *LINC01116* compared to the normal samples using the GSE45827 microarray dataset. There was no significant change in the *LINC01116* expression in all tumor samples. However, the results revealed a significant upregulation and downregulation of the *LINC01116* expression in ER+ and ER− samples, respectively (Figure 2A). Additionally, the *LINC01116* expression pattern in different breast cancer subtypes was assessed. The results showed *LINC01116*, as compared to the normal samples, significantly elevated in Luminal A and B, meaningfully reduced in TNBC, and not changed in HER2+ samples (Figure 2B).

Moreover, the expression level of *LINC01116* in breast tumor versus tumor margins tissues was evaluated using qRT‐PCR. There was no significant change in *LINC01116* expression in all tissues and ER+ samples compared to tumor margin tissues. Nevertheless, significant downregulation of the *LINC01116* expression was observed in ER− samples (Figure 2C). In addition, we examined the expression of *LINC01116* in lymph node statuses and tumor grades among ER+ samples. The data showed significant *LINC01116* downregulation in N2 and N3, compared with N1 tissues (Figure 2D), and in grades II as well as III, compared to grade I (Figure 2E), suggesting the reduction of *LINC01116* expression is related to tumor development in ER+ samples.

### 3.3 *LINC01116* differentially affects patients' survival

**FIGURE 3:**
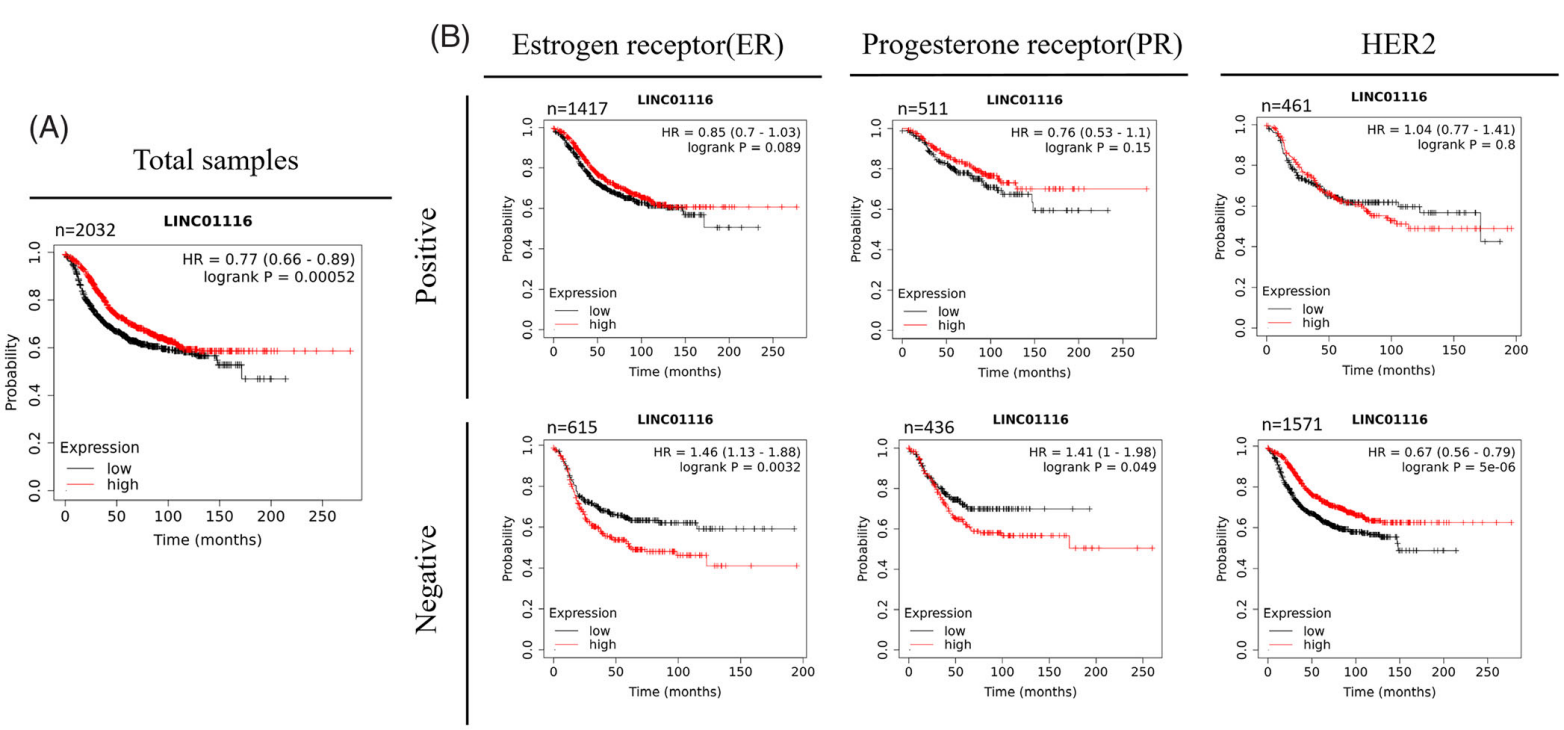
Relationship between *LINC01116* expression and RFS in breast cancer patients (based on microarray meta‐analysis). Kaplan–Meier survival curve for *LINC01116* in all samples (*n* = 2032) indicates that breast cancer patients with high expression levels of *LINC01116* have more survival chance. *p*‐value = .00052 (A). The Kaplan–Meier survival curves for *LINC01116* in separated tissues based on the status of hormone receptors. While high‐level expression of *LINC01116* confers better survival chance in HER2‐ breast cancer patients (*n* = 1571, *p*‐value = .000005), ER− (*n* = 615) and PR− (*n* = 436) breast cancer patients with high expression levels of *LINC01116* have a lower chance of survival. *p*‐value = .0032 for ER− and *p*‐value = .049 for PR− (B).

**FIGURE 4:**
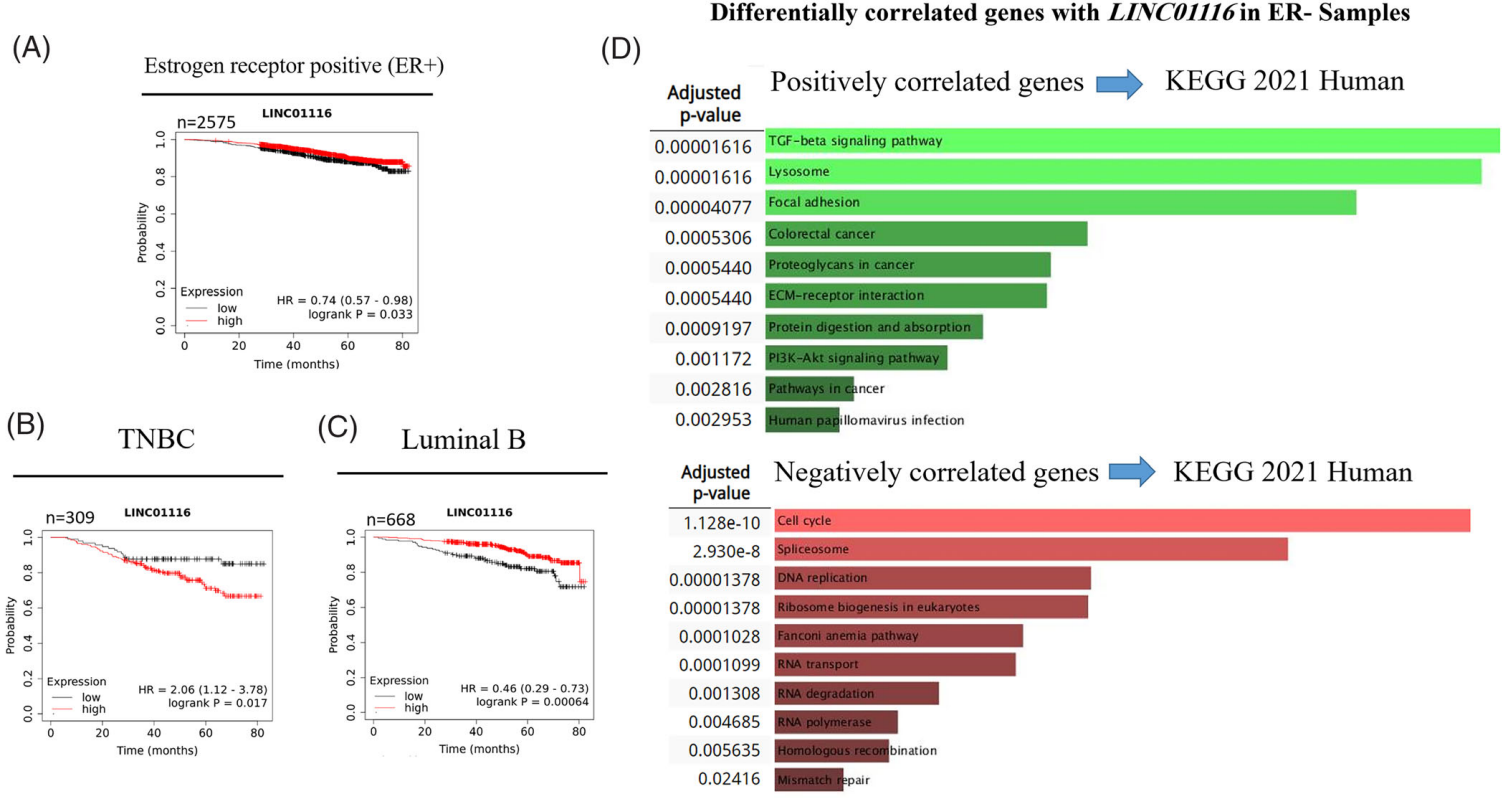
Relationship between *LINC01116* expression and overall survival in breast cancer patients (based on RNA‐seq meta‐analysis). Kaplan–Meier survival curve for *LINC01116* in ER+ samples (*n* = 2575) indicates that the high expression level of *LINC01116* increases the survival rate of the patients. *p*‐value = .033 (A). Although the expression level of *LINC01116* negatively correlates with the survival rate of TNBC breast cancer patients (*n* = 309) (*p*‐value = .017) (B), there is a positive correlation between the expression of *LINC01116* and the survival chance of Luminal B breast cancer patients (*p*‐value = .00064) (C). KEGG 2021 Human pathway analysis for the differentially correlated genes in ER− samples. The positively correlated genes with *LINC01116* were mainly involved in the TGF‐β signaling pathway (Adj. *p*‐value = .00001616), and the negatively correlated genes with *LINC01116* were mostly involved in the cell cycle (Adj. *p*‐value = 1.128e‐10) (D).

Meta‐analysis of microarray data in the KM plotter was performed to investigate the impact of *LINC01116* on patient survival probability. It was revealed that the expression of *LINC01116* meaningfully correlates with a better survival rate in all patients with breast cancer (Figure 3A). Surprisingly, it was shown that *LINC01116* in ER− or Progesterone‐receptor‐negative (PR−) tissues significantly correlates with the low chance of survival in patients. In addition, the expression level of *LINC01116* in HER2‐ tissue samples positively correlates to patients' survival rate (Figure 3B). Although microarray data analysis did not show meaningful survival related to *LINC01116* expression in the ER+ and Progesterone‐receptor‐positive (PR+) samples (Figure 3B), Ref‐seq mRNA analysis revealed a significant positive correlation between *LINC01116* expression and survival rate in ER+ samples (Figure 4A). Additionally, since HER2‐ samples, principally based on ER status, divide into Luminal A and B or TNBC, Ref‐seq mRNA survival analysis was used to determine the impact of ER+ and PR+ in HER2‐ samples on the relation between *LINC01116* and patients' survival. The results exhibited a meaningful negative correlation in TNBC (Figure 4B), a significant positive correlation in Luminal B (Figure 4C), and no significant correlation in Luminal A (data not shown) between *LINC01116* expression and patients' survival, suggesting survival effect of *LINC01116* is affected by ER (and probably PR) status of samples. Due to the different impact of *LINC01116* on survival rates in ER+ and ER− tissues, differentially correlated genes with *LINC01116* in these samples were collected in Table 3 (*p*‐value<.02 and the sum of the absolute value of the correlations >.7). Also, all genes meaningfully correlated with *LINC01116* in ER+ and ER− samples are listed in the supplementary material Table S3.

**TABLE 3:** Genes that are negatively correlated with *LINC01116* in ER+ samples and positively correlated in ER− samples (the sum of the absolute value of the correlations >.7) (*p*‐value<.02).

Gene symbol	Official full name	Gene ID	Correlation with LINC01116 among ER+ samples	Correlation with LINC01116 among ER− samples	The sum of the absolute value of the correlations	Action in breast tumors	References
*HIF1A*	hypoxia inducible factor 1 subunit alpha	3091	−.35	.53	.88	Oncogene, maintenance of cancer cell stemness, correlation with poor prognosis, and related to the drug resistance or drug‐low‐efficacy (Tamoxifen, Trastuzumab, 5‐Fluorouracil, Fulvestrant, Gemcitabine, Letrozole, and Methotrexate)	60
*STS*	steroid sulfatase	412	−.46	.39	.85	Contribute to the growth and development of breast cancer, a possible pharmacological target for the treatment of malignancies and Tamoxifen resistance	61, 62
*SLC16A14*	solute carrier family 16 member 14	151 473	−.41	.43	.84	Upregulate in both Tamoxifen resistance MCF7 cell line and patients with incomplete response to chemotherapy	63
*MSX2*	msh homeobox 2	4488	−.36	.47	.83	Reduce Tamoxifen resistance and promote apoptosis (anti‐mitogenic role) Induce EMT and closely associate with bone marrow metastasis (mitogenic role)	64, 65
*ALDH3B2*	aldehyde dehydrogenase 3 family member B2	222	−.38	.44	0.81	Contribute to poor prognosis, related to bone marrow metastasis and Tamoxifen resistance, a potential cancer stem cell marker in HER2+ cells	66, 67, 68
*CAMK2N1*	calcium/calmodulin dependent protein kinase II inhibitor 1	55 450	−.35	.44	.79	Link to the development of invasive breast cancer from Ductal Carcinoma In Situ (DCIS), associated with poor prognosis in ER− samples (supplementary material Figure 1A)	69
*C3orf14*	chromosome 3 open reading frame 14	57 415	−.36	.40	.76	Upregulate in Trastuzumab resistance patients compared to responsive ones	70
*PGAP3*	post‐GPI attachment to proteins phospholipase 3	93 210	−.30	.45	.75	Promote growth and metastasis in TNBC	71
*REEP3*	receptor accessory protein 3	221 035	−.31	.43	.74	Poorly studied in breast cancer	
*ATP2C2*	ATPase secretory pathway Ca2+ transporting 2	9914	−.40	.34	.74	Negatively correlate with patient survival, promote tumorigenesis, suppress EMT in TNBC, and can drive chemotherapy resistance	72, 73, 74, 75, 76
*SEC16A*	SEC16 homolog A, endoplasmic reticulum export factor	9919	−.39	.35	.74	Poorly studied in breast cancer Nonetheless, it is reported that there are SEC16A‐NOTCH1 fusions in breast cancer, causing NOTCH1 signaling.	77
*ACVR1B*	activin A receptor type 1B (also known as ALK4)	91	−.32	.40	.72	Downregulation of *ACVR1B* inhibits cell proliferation and survival as well as angiogenesis.	78
*ARFGEF3*	ARFGEF family member 3 (also known as BIG3)	57 221	−.33	.39	.72	Correlate with poor prognosis and lead to Tamoxifen resistance by deactivating Prohibitin 2 (*PHB2*)	79, 80
*RP2*	RP2 activator of ARL3 GTPase	6102	−.37	.33	.70	Upregulate in breast cancer, link to poor survival in ER+ patients (material mentary data Figure 1B)	81

### 3.4 The overexpression of *LINC01116* induces TGF‐β signaling

To determine the pathways in which *LINC01116* is involved – in ER− samples, we collected negatively and positively correlated genes with *LINC01116* using GSE45827 microarray data analysis (*p*‐value <.05). Enrichr online tool (using the KEGG pathway analysis) revealed that positively correlated genes were mainly involved in the TGF‐β signaling pathway, and negatively correlated genes were primarily involved in the cell cycle (Figure 4D).

**FIGURE 5:**
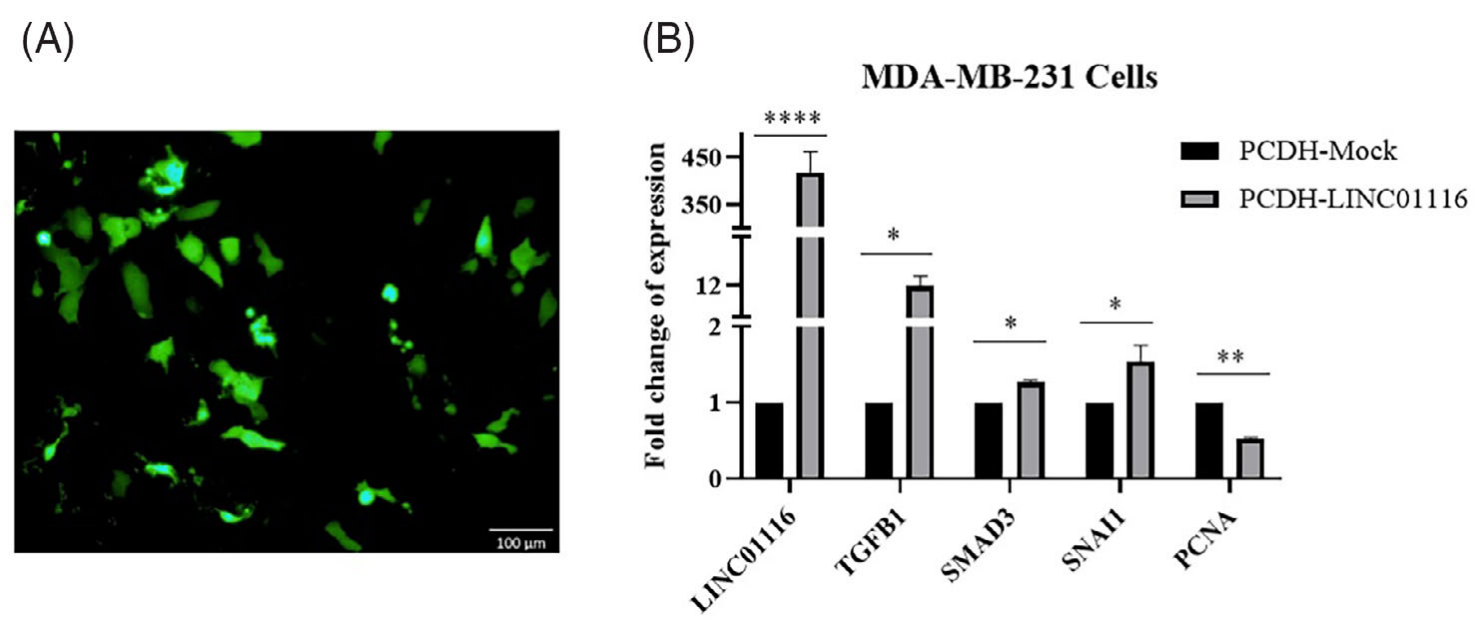
The overexpression of *LINC01116* induces TGF‐β signaling in MDA‐MB‐231 cells. Green fluorescence indicating transfection of PCDH‐LINC01116 in MDA‐MB‐231 cells (A), qRT‐PCR analysis shows mRNA expression levels of *TGFB1*, *SMAD3*, *SNAI1*, and *PCNA* in MDA‐MB‐231 cells. The *β2m* was used as the internal control. Values are presented as the mean ± SD of three independent replicates. ****p‐value<.0001, **p‐value<.01, *p‐value<.05 (B).

Moreover, to examine the effect of *LINC01116* on TGF‐β signaling, a gain of function approach was employed in MDA‐MB‐231 cells. To this aim, transient MDA‐MB‐231cells overexpressing *LINC01116* were generated (Figure 5A). The qRT‐PCR analysis showed successful upregulation of *LINC01116* in the transfected cells. In addition, our results showed a significant upregulation in the transforming growth factor beta 1 (*TFGB1*) and its downstream genes, such as SMAD family member 3 (*SMAD3*) and snail family transcriptional repressor 1 (*SNAI1*) in MDA‐MB‐231 PCDH‐LINC01116 transfected cells compared to the PCDH‐Mock transfected ones. Moreover, we measure the expression of the proliferating cell nuclear antigen (*PCNA*) as a marker for proliferation^44^ to examine the effect of *LINC01116* overexpression on the proliferation of the MDA‐MB‐231 cells. The result exhibited a significant downregulation of *PCNA* in PCDH‐LINC01116 transfected MDA‐MB‐231 cells compared to the Mock ones (Figure 5B).

## 4 DISCUSSION

Breast cancer is the most frequently diagnosed cancer and one of the top causes of cancer death worldwide.^45^ Early diagnosis of breast tumors and detection of breast cancer subtypes can significantly lead to more successful treatment outcomes and an increased survival rate.^3^, ^4^, ^46^ lncRNAs can serve as a potential diagnostic biomarker for breast cancer.^9^ Although numerous types of research have been dedicated to the functions of lncRNAs to discover novel targets for the detection and treatment of breast cancer,^47^, ^48^, ^49^ the therapeutic and diagnostic approaches available to breast cancer patients are less than satisfactory.

Previous studies on the *LINC01116* role in ischemic injuries and cancer disease suggest that *LINC01116* can perform diverse functions depending on different molecular contexts. For instance, it has been demonstrated that *LINC01116* has contradictory effects on cell death and AKT signaling in different cells.^19^, ^20^, ^21^, ^22^, ^23^, ^24^, ^25^, ^26^, ^27^ Although the role of *LINC01116* has been investigated in different studies on several types of cancer,^19^, ^20^, ^21^, ^22^, ^23^, ^24^ there are limited studies about its role in breast cancer. A study has shown that *LINC01116* is upregulated in both breast cancer samples and breast cancer cell lines, especially in ER+ cells like the MCF7cell line, compared with the TNBC cell line (MDA‐MB‐231) and normal cells (MCF10A).^24^ In accordance with previous research findings, the present study provides evidence that the expression of *LINC01116* is markedly elevated in ER+ breast cancer tissues. On the other hand, our result reveals a significant downregulation of the *LINC01116* expression in ER− ones. Moreover, for the first time, we reveal that *LINC01116* can serve as a potential biomarker to distinguish ER+ and ER− breast cancer subtypes. Additionally, our results suggest that *LINC01116* expression positively correlates with the survival rate in ER+ breast cancer patients. This pattern has also been shown in a previous study in kidney renal clear cell carcinoma.^50^ Nonetheless, the correlation between *LINC01116* and survival rate is negative in ER− breast cancer cohort, which aligns with previous research on glioma and lung adenocarcinoma.^19^, ^21^

Estrogen signaling is one of the most crucial signaling pathways in breast cancer, which mediates cell proliferation and apoptosis and suppresses EMT.^13^, ^14^, ^15^ ER is a desired target for endocrine therapy and confers a better prognosis in breast cancer patients.^51^ Our results show that the expression of *LINC01116* is upregulated in 17β‐Estradiol treated MCF7 cells, and there is a direct relation between *LINC01116* expression and ER signaling. Consequently, ER signaling seems responsible for the elevated *LINC01116* expression in ER+ samples compared to ER− ones. Likewise, a study by Hu et al. showed a positive correlation between *LINC01116* and Estrogen receptor 1 (*ESR1*) in breast cancer samples, and they showed that *LINC01116* acts as a competitive endogenous RNA (ceRNA) to regulate *ESR1* expression by competing with miR‐145.^24^ In addition, previous studies demonstrated that *ESR1* positively correlates with the survival rate in breast cancer patients,^51^ and silencing *ESR1* induces EMT and endocrine drug resistance,^15^, ^52^ Supporting the results of this study that *LINC01116* positively correlates with better survival rate in ER+ breast cancer.

Previous studies showed that the expression of *LINC01116* is upregulated in Gefitinib resistance non‐small lung cancer,^53^ Doxorubicin resistance osteosarcoma,^54^ and Cisplatin resistance lung adenocarcinoma cells.^55^ Surprisingly, our results showed that the *LINC01116* expression is decreased in Tamoxifen‐resistant MCF7 cells compared to Tamoxifen‐sensitive cells. Since the Tamoxifen resistance cells show metastatic and invasive phenotypes,^56^ it seems that the downregulation of *LINC01116* correlates with a poor survival rate in ER+ breast cancer. Furthermore, our results in ER+ samples demonstrate that by developing lymph nodes and grade states, the expression of *LINC01116* is reduced. In addition, among these samples, *LINC01116* negatively correlates with genes involved in drug resistance (*HIF1A*, *STS*, *SLC16A14*, *ALDH3B2*, *ATP2C2*, and *ARFGEF3*) (Table 3), suggesting that *LINC01116* may confer satisfactory survival chance in ER+ patients.

The TGF‐β function in cancer is relatively complicated. Although the TGF‐β is antiproliferative, it can promote EMT, drug resistance, and invasion in breast cancer cells.^57^, ^58^ A previous study demonstrated that the knockdown of *LINC01116* inhibits the TGF‐β signaling via the miR‐744‐5p axis in glioma.^19^ In the present study, we show that the overexpression of *LINC01116* increases the TGF‐β signaling in MDA‐MB‐231 cells and upregulates the *SNAI1* expression, which acts as a marker for EMT.^14^ In addition, apart from the positive correlation between *LINC01116* and the aforementioned genes involved drug resistance in ER− samples (Table 3), there is a positive correlation between *LINC01116* and Activin A Receptor Type 1B (*ACVR1B*, also known as *ALK4*), which is related to the TGF‐β superfamily and promotes invasion, EMT, and metastasis in breast cancer.^59^ All these findings confirm the negative effect of *LINC01116* on ER− patients' survival.

To conclude, our results demonstrate that *LINC01116* is differentially expressed in breast cancer based on ER status. For the first time, we also suggest that *LINC01116* can be a potential biomarker to distinguish between ER+ and ER− samples. Furthermore, our data revealed that *LINC01116* affects patients' survival differentially, by involving in the ER and TGF‐β signaling pathways, depending on the ER status. The result of this study can be utilized to develop biomarkers to distinguish ER+ from ER− breast cancer and better understand the molecular mechanism underlying breast cancer subtypes. Some limitations should be admitted while the provided data are analyzed. First, the number of breast cancer samples could be more to introduce *LINC01116* as a more promising biomarker. Second, the role of *LINC01116* in TGF‐β signaling in ER+ breast cancer cells could be investigated by performing a loss of function assay. For this reason, we recommend further studies to elucidate the precise *LINC01116* roles in crosstalk TGF‐β and ER signaling in ER+ subtypes and illuminate the effect of *LINC01116* in drugs resistance, particularly Tamoxifen resistance in breast cancer cells.

## AUTHOR CONTRIBUTIONS

**Mohammadjavad Karimi Taheri:** Formal analysis (equal); investigation (equal); resources (equal); software (equal); validation (equal); visualization (equal); writing – original draft (equal); writing – review and editing (equal). **Sogol Ghanbari:** Formal analysis (equal); investigation (equal); resources (equal); validation (equal); writing – original draft (equal); writing – review and editing (equal). **Akram Gholipour:** Formal analysis (equal); investigation (equal); writing – review and editing (equal). **Taraneh Givi:** Formal analysis (equal); investigation (equal); validation (equal); writing – review and editing (equal). **Majid Sadeghizadeh:** Conceptualization (lead); investigation (equal); project administration (lead); supervision (lead); writing – review and editing (equal).

## FUNDING STATEMENT

This research did not receive any specific grant from funding agencies in the public, commercial, or not‐for‐profit sectors.

## CONFLICT OF INTEREST STATEMENT

The authors have no relevant financial or non‐financial interests to disclose.

## ETHICS APPROVAL STATEMENT

This research was performed in accordance with the Ferdowsi University of Mashhad Ethics Committee (code number: R.UM.REC.1399.104).

## PATIENT CONSENT STATEMENT

The patients provided signed written informed consent after receiving extensive exposure to the purpose of the study.

## Supporting information


**FIGURE S1.** Kaplan–Meier survival curves for *CAMK2N1* in ER− samples (A) and *RP2* in ER+ samples (B).

**TABLE S1.** Pathological and clinical data of tumor specimens. T; tumor size, N; lymph node status, M; distant metastases (according to the TNM classification system), ER; Estrogen receptor, PR; Progesterone receptor, HER2; Human epidermal growth factor receptor 2, N/A; not available.

**TABLE S2.** The sequence of qRT‐PCR Primers.

**TABLE S3.** The genes correlated (Pearson method) with *LINC01116* in ER+ samples.

## Data Availability

All data generated or analyzed during this study are included in this published article [and its supplementary material files].
