# Supplementary figures and images for: LINC01116 affects patient survival differently and is dissimilarly expressed in ER+ and ER− breast cancer samples
Source: Cancer Rep (Hoboken). 2023 Jun 15;6(8):e1848. doi: 10.1002/cnr2.1848 (PMC10432450; doi:10.1002/cnr2.1848)

A

Estrogen receptor negative (ER-)

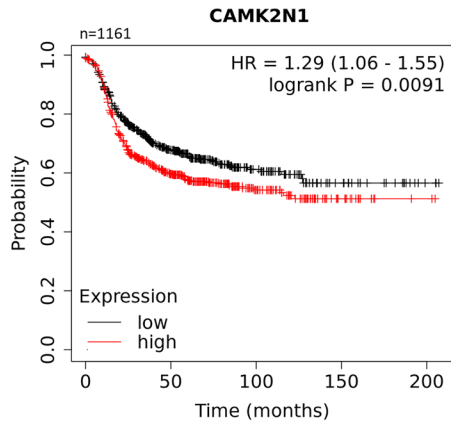

B

Estrogen receptor positive (ER+)

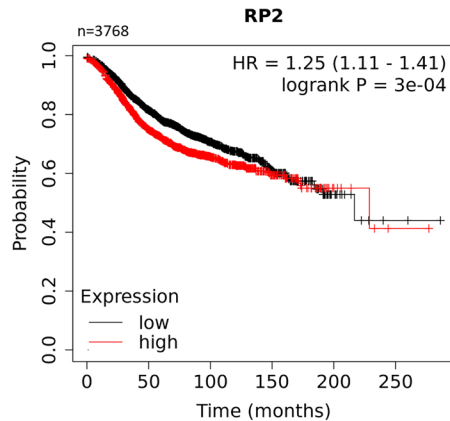

Supplement: Supplementary file 1 — FIGURE S1. Kaplan–Meier survival curves for CAMK2N1 in ER− samples (A) and RP2 in ER+ samples (B). [file CNR2-6-e1848-s004.pdf]
