# Supplementary material for: LINC01116 affects patient survival differently and is dissimilarly expressed in ER+ and ER− breast cancer samples
Source: Cancer Rep (Hoboken). 2023 Jun 15;6(8):e1848. doi: 10.1002/cnr2.1848 (PMC10432450; doi:10.1002/cnr2.1848)
Supplement: Supplementary file 2 — TABLE S1. Pathological and clinical data of tumor specimens. T; tumor size, N; lymph node status, M; distant metastases (according to the TNM classification system), ER; Estrogen receptor, PR; Progesterone receptor, HER2; Human epidermal growth factor receptor 2, N/A; not available. [file CNR2-6-e1848-s002.docx]

**Supplementary material Table 1. Pathological and clinical data of tumor specimens.**

| Patient Number | Patient age | ER | PR | HER2 | T | N | M | Tumor Grade | Tumor Stage |
| --- | --- | --- | --- | --- | --- | --- | --- | --- | --- |
| 1 | **41** | **-** | **-** | **-** | **2** | **1** | **0** | **2** | **2B** |
| 2 | **48** | **+** | **+** | **+** | **N/A** | **N/A** | **0** | **N/A** | **N/A** |
| 3 | **58** | **+** | **+** | **+** | **2** | **0** | **0** | **2** | **2A** |
| 4 | **60** | **-** | **-** | **+** | **2** | **0** | **0** | **2** | **2A** |
| 5 | **26** | **-** | **-** | **-** | **2** | **1** | **0** | **3** | **2B** |
| 6 | **42** | **-** | **-** | **-** | **2** | **2** | **0** | **3** | **3A** |
| 7 | **65** | **+** | **+** | **-** | **1** | **0** | **0** | **1** | **1** |
| 8 | **65** | **-** | **-** | **N/A** | **N/A** | **N/A** | **0** | **N/A** | **N/A** |
| 9 | **52** | **-** | **-** | **+** | **2** | **2** | **0** | **2** | **2A** |
| 10 | **50** | **+** | **+** | **-** | **2** | **1** | **0** | **2** | **2B** |
| 11 | **47** | **-** | **-** | **N/A** | **2** | **0** | **0** | **3** | **2A** |
| 12 | **46** | **+** | **+** | **-** | **2** | **0** | **0** | **3** | **2A** |
| 13 | **35** | **+** | **+** | **+** | **2** | **0** | **0** | **2** | **2A** |
| 14 | **66** | **+** | **-** | **-** | **2** | **0** | **0** | **2** | **2A** |
| 15 | **63** | **-** | **-** | **-** | **1** | **0** | **0** | **3** | **1** |
| 16 | **47** | **-** | **-** | **-** | **1** | **0** | **0** | **3** | **1** |
| 17 | **35** | **+** | **+** | **-** | **N/A** | **N/A** | **0** | **3** | **N/A** |
| 18 | **37** | **+** | **+** | **-** | **2** | **0** | **0** | **2** | **2A** |
| 19 | **45** | **+** | **+** | **-** | **1** | **1** | **0** | **1** | **2A** |
| 20 | **60** | **+** | **+** | **-** | **1** | **0** | **0** | **2** | **1** |
| 21 | **53** | **+** | **N/A** | **-** | **3** | **0** | **0** | **2** | **2B** |
| 22 | **46** | **+** | **+** | **-** | **2** | **1** | **0** | **2** | **2B** |
| 23 | **50** | **+** | **+** | **-** | **1** | **3** | **0** | **2** | **3C** |
| 24 | **58** | **-** | **-** | **+** | **1** | **0** | **0** | **N/A** | **1** |
| 25 | **45** | **+** | **+** | **-** | **N/A** | **N/A** | **N/A** | **N/A** | **N/A** |
| 26 | **50** | **+** | **+** | **-** | **1** | **1** | **0** | **2** | **2A** |
| 27 | **41** | **+** | **+** | **-** | **1** | **0** | **0** | **2** | **1** |
| 28 | **55** | **+** | **+** | **-** | **1** | **0** | **0** | **2** | **1** |
| 29 | **40** | **+** | **+** | **-** | **1** | **0** | **0** | **2** | **1** |
| 30 | **42** | **+** | **+** | **-** | **1** | **1** | **0** | **2** | **2A** |
| 31 | **67** | **+** | **+** | **-** | **2** | **0** | **0** | **2** | **2A** |
| 32 | **39** | **+** | **+** | **-** | **3** | **2** | **0** | **3** | **3A** |
| 33 | **55** | **+** | **+** | **-** | **1** | **0** | **0** | **2** | **1** |
| 34 | **72** | **+** | **+** | **-** | **2** | **0** | **0** | **2** | **2A** |
| 35 | **36** | **+** | **+** | **-** | **2** | **1** | **0** | **3** | **2B** |
| 36 | **61** | **+** | **+** | **-** | **2** | **2** | **0** | **2** | **3A** |
| 37 | **50** | **+** | **+** | **-** | **N/A** | **N/A** | **0** | **3** | **N/A** |
| 38 | **47** | **+** | **+** | **-** | **3** | **0** | **0** | **1** | **2B** |
| 39 | **49** | **+** | **+** | **-** | **1** | **2** | **0** | **1** | **3A** |
| 40 | **50** | **-** | **-** | **-** | **N/A** | **N/A** | **0** | **N/A** | **N/A** |

T; tumor size, N; lymph node status, M; distant metastases (according to the TNM classification system), ER; estrogen receptor, PR; progesterone receptor, HER2; human epidermal growth factor receptor 2, N/A; not available.
