# Supplementary material for: LINC01116 affects patient survival differently and is dissimilarly expressed in ER+ and ER− breast cancer samples
Source: Cancer Rep (Hoboken). 2023 Jun 15;6(8):e1848. doi: 10.1002/cnr2.1848 (PMC10432450; doi:10.1002/cnr2.1848)
Supplement: Supplementary file 3 — TABLE S2. The sequence of qRT‐PCR Primers. [file CNR2-6-e1848-s001.docx]

**Supplementary material Table 2. The sequence of qRT-PCR Primers.**

| Primer Sequence (5'-3') | Amplicon size (bp) | Accession number | Gene |
| --- | --- | --- | --- |
| Forward: AGGAAATGACCCGAACTGCCAG  Reverse: ATATTGAACTGAGCGGGGCTTTCG | 120 | ENSG00000163364 | *LINC01116* |
| Forward: CGACTGCGGATCTCTGTGC  Reverse: CAGTAGTGTTCCCCACTGGTC | 161 | ENSG00000105329 | *TGFB1* |
| Forward: GGAGGAGAAATGGTGCGAGAAG  Reverse: CACAGGCGGCAGTAGATGAC | 198 | ENSG00000166949 | *SMAD3* |
| Forward: CGGAAGCCTAACTACAGCGAG  Reverse: AGATGAGCATTGGCAGCGAG | 139 | ENSG00000124216 | *SNAI1* |
| Forward: AGCACCAAACCAGGAGAAAGT  Reverse: TCACTCCGTCTTTTGCACAG | 191 | ENSG00000132646 | *PCNA* |
| Forward: CTACTCTCTCTTTCTGGCCTG  Reverse: GACAAGTCTGAATGCTCCAC | 191 | ENSG00000166710 | *β2M* |
